# Cataracte rompue post traumatisme contusif

**DOI:** 10.11604/pamj.2014.17.279.4193

**Published:** 2014-04-14

**Authors:** Salim Belhassan, Rajae Daoudi

**Affiliations:** 1 Université Mohammed V Souissi, Service d'Ophtalmologie A de l'hôpital des spécialités, Centre Hospitalier Universitaire, Rabat, Maroc

**Keywords:** Cataracte, contusion, œil, cataract, contusion, eye

## Image en medicine

Patient âgé de 29 ans, sans antécédents particuliers qui a présenté suite à un syndrome contusif (agression par coup de poing) au niveau de l’œil gauche, une baisse de l'acuité visuelle. L'examen ophtalmologique trouve une acuité visuelle à compte les doigts de près au niveau de l’œil atteint, à la lampe à fente on trouve une cornée claire, une bonne chambre antérieure avec une cataracte rompue en chambre antérieure et un tonus oculaire à 16mmHg. L'examen du fond d’œil était inaccessible et l’échographie oculaire n'a pas objectivé de décollement de rétine. L’œil Adelphe était sans particularités. Ce patient doit bénéficier d'une Phaco émulsification extra capsulaire avec mise en place d'un implant intra oculaire dans le sac postérieur si possible, s'il n'y a pas moyen d'implantation de le sac à ce moment-là une implantation en chambre antérieure ou à fixation sclérale est envisageable.

**Figure 1:**
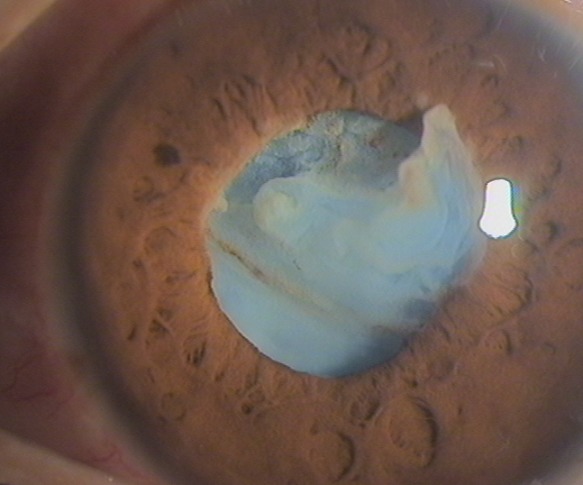
Image caractéristique de la cataracte post traumatique

